# Residual-guided hybrid framework for adversarially robust deep learning-based network intrusion detection

**DOI:** 10.1371/journal.pone.0350737

**Published:** 2026-06-01

**Authors:** Sudip Saha, Muhammad Arslan Pervaiz, Muhammad Safwat Rahman, Shahriar Ahmmed, Jannatul Maua

**Affiliations:** 1 Department of Cybersecurity, Pace University, New York, New York, United States of America; 2 Department of Computer Science, Southeast Missouri State University, Cape Girardeau, Missouri, United States of America; 3 Department of ICT, Govt. Shah Sultan College, Bogura‌‌, Bangladesh; 4 Department of Computer Science and Engineering, Bangladesh University of Business and Technology, Dhaka, Bangladesh; The University of Aukland, NEW ZEALAND

## Abstract

The growing sophistication of cyber threats and adversarial attacks poses critical challenges to the security and robustness of machine learning models deployed in real-world systems. While traditional deep learning architectures excel in clean data classification, they often fail under adversarial perturbations, exposing vulnerabilities in sensitive domains such as healthcare, finance, and industrial control. In this paper, we introduce a novel hybrid adversarially-trained deep learning framework that integrates reinforcement learning-inspired robustness adaptation with knowledge-driven regularization to achieve improved resilience against fast gradient sign method (FGSM) and projected gradient descent (PGD) attacks. Our approach simultaneously optimizes clean accuracy and adversarial robustness by balancing cross-entropy and adversarial loss components, while monitoring calibration error, gradient dynamics, and generalization gap to ensure stable convergence. Extensive experiments on reconnaissance, shellcode, and worms datasets demonstrate that the proposed model achieves up to 97.88% accuracy on clean data and maintains 84.9% accuracy under FGSM and 81.75% under PGD attacks, outperforming convolutional neural network (CNN) and long short-term memory (LSTM) baselines by more than 6–10 percentage points in adversarial robustness. Furthermore, the training curves reveal consistent improvements in convergence stability, runtime efficiency, gradient norm decay, and a 30% reduction in expected calibration error, validating the scalability of the framework. This work contributes not only a novel adversarial defense paradigm but also provides insights into the trade-offs between robustness, efficiency, and generalization.

## 1 Introduction

In recent years, the rapid growth of artificial intelligence and deep learning has transformed a wide range of domains, from computer vision and natural language processing to cybersecurity and healthcare analytics, including network intrusion detection systems (NIDS) [1]. Despite these advances, it has become increasingly evident that modern machine learning models are not immune to vulnerabilities, particularly when faced with adversarial perturbations, distributional shifts, or noisy environments [2]. These shortcomings not only limit the reliability of deep models in real-world scenarios but also pose serious risks when applied in safety-critical systems such as medical diagnostics, autonomous vehicles, and financial decision-making [3,4]. As a result, the development of robust, reliable, and generalizable learning frameworks has emerged as a pressing challenge within the broader field of trustworthy artificial intelligence [5].

Against this backdrop, the specific research problem addressed in this work centers on the need for models that can simultaneously achieve robustness against adversarial disturbances, maintain high predictive accuracy on clean data, and generalize effectively across diverse and dynamic environments [6]. Traditional approaches, such as adversarial training and robust optimization, have laid important foundations but often encounter limitations, including excessive computational costs, reduced clean-data performance, and weak transferability across domains [7].

The motivation for this research stems from the recognition that the current state of adversarial defense strategies remains fragmented, with most techniques optimized for isolated objectives. Our work aims to bridge this gap by proposing a novel model that integrates the strengths of deep learning architectures with carefully designed training strategies, resulting in a framework that can resist adversarial attacks, improve calibration, and ensure generalization in practical applications [8]. The purpose of this study is therefore twofold: first, to introduce a principled methodology that addresses the shortcomings of existing approaches, and second, to provide a comprehensive evaluation that demonstrates the broader applicability of the proposed solution across diverse datasets and experimental conditions [9].

The significance of this research lies in its potential to enhance the reliability and trustworthiness of machine learning systems. By achieving robustness without sacrificing performance or scalability, the proposed framework offers new opportunities for deploying AI in domains where failure tolerance is minimal and trust in automated decisions is essential. Moreover, the implications of this work extend beyond technical robustness, contributing to the broader discourse on ethical and responsible AI by emphasizing transparency, adaptability, and resilience [10].

To realize these objectives, we design a hybrid adversarial learning framework that combines multi-stage training procedures with adaptive loss formulations and architecture-level refinements. Our approach is evaluated using benchmark datasets under a wide spectrum of attack scenarios, ensuring that the results capture both adversarial resistance and generalization capabilities. Through systematic experimentation, we demonstrate the superiority of our method in balancing robustness, efficiency, and calibration compared to existing baselines.

### Contributions of this study

This work proposes a residual-guided hybrid defense framework designed to improve adversarial robustness in deep learning-based network intrusion detection systems. The main contributions include: (i) integrating residual-based adversarial detection with adversarially trained classification in a unified architecture, (ii) improving robustness while preserving clean-data accuracy and calibration reliability, (iii) providing extensive experimental validation across multiple attack categories and adversarial settings, and (iv) analyzing training dynamics, robustness behavior, and generalization characteristics to better understand the stability of adversarially robust learning systems.

The key contributions of this paper can be summarized as follows:

- We introduce a novel hybrid framework that unifies adversarial robustness, calibration, and generalization within a single training paradigm.
- We propose an adaptive optimization strategy that reduces the trade-off between robustness and clean-data accuracy while maintaining computational efficiency.
- We provide an extensive evaluation across multiple datasets and attack settings, highlighting the versatility and scalability of the proposed method.
- We analyze the theoretical underpinnings of the framework, offering insights into its stability, convergence properties, and implications for trustworthy AI.

The remainder of the paper is structured as follows: Section II reviews the related work in adversarial robustness and defense strategies. Section III details the proposed methodology, including the model architecture, training pipeline, and optimization techniques. Section IV reports and analyzes the results, while Section V provides a detailed discussion of the findings, limitations, and future directions. Finally, Section VI concludes the paper with a summary of contributions and implications. All the abbreviations used in the paper are presented in Table 1.

**Table 1:** List of abbreviations used in this manuscript.

Abbreviation	Definition
NIDS	Network Intrusion Detection System
FGSM	Fast Gradient Sign Method
PGD	Projected Gradient Descent
DAE	Denoising Autoencoder
ZCA	Zero-phase Component Analysis
CE	Cross-Entropy
IQR	Interquartile Range
MAD	Median Absolute Deviation
ROC	Receiver Operating Characteristic
PR	Precision–Recall
AUC	Area Under the Curve
ROC-AUC	Area Under the ROC Curve
PR-AUC	Area Under the PR Curve
ECE	Expected Calibration Error
ASR	Attack Success Rate
ADR	Adversarial Detection Rate
SVM	Support Vector Machine
CNN	Convolutional Neural Network
LSTM	Long Short-Term Memory

## 2 Related work

Research on adversarial robustness and generalization has evolved rapidly over the past decade and has seen significant advances in recent years (2024–2026), driven by the recognition that deep learning models, despite their remarkable predictive performance, remain vulnerable to subtle perturbations that can mislead them with high confidence. Early studies on adversarial attacks established that neural networks exhibit inherent fragility against gradient-based perturbations, prompting the development of defense mechanisms that aimed to enhance robustness through training-time strategies, model regularization, and architectural innovations [11,12]. Adversarial training quickly emerged as the most widely studied defense, in which models are explicitly trained with adversarially perturbed samples to harden their decision boundaries [7]. While effective, this approach is often accompanied by a trade-off in clean-data accuracy and increased computational cost, motivating recent research on improving training efficiency and sample selection strategies for adversarial learning [13]., and subsequent research has sought to strike a balance between robustness and generalization by introducing regularization techniques, data augmentation strategies, and curriculum-based adversarial schedules [14].

Beyond adversarial training, other strands of work have explored robust optimization, adversarial detection mechanisms, and structured defense frameworks designed to identify malicious perturbations during inference [15]. These efforts include margin-based defenses, stability-driven training schemes, and gradient masking techniques, each with varying degrees of success and limitations [16]. Parallel to these developments, significant attention has been devoted to calibration and the reliability of model confidence scores, as robust models must not only resist adversarial perturbations but also produce well-calibrated probability estimates [17]. Poor calibration can undermine trust in predictions, particularly in safety-critical applications such as healthcare, finance, and autonomous systems. Several studies have therefore sought to unify robustness with calibration improvements, highlighting the interconnectedness of these two objectives [18].

In addition, the landscape of adversarial robustness research has expanded into different modalities and domains. Beyond computer vision, work on adversarial defenses has extended to natural language processing, speech recognition, and graph-based learning, where models face unique challenges due to discrete structures, sequential dependencies, and relational information [19–21]. At the same time, efforts have been made to develop more efficient adversarial defenses that reduce computational costs, making them more suitable for deployment in real-world settings where resources are constrained. The intersection of adversarial robustness with explainability, fairness, and energy efficiency has also gained momentum, underscoring the multidimensional nature of trustworthy machine learning and the need for holistic defense strategies [22].

Recent advances have further examined the role of architectural design, optimization dynamics, and loss function engineering in achieving robustness without sacrificing scalability. Models with specialized layers, regularization penalties, and training heuristics have been proposed to stabilize gradient behavior and improve resistance to attacks [23]. Similarly, studies on the generalization gap between training and adversarial performance have motivated adaptive learning frameworks that dynamically adjust to evolving threat models. Despite these efforts, challenges remain in achieving robustness that generalizes across datasets, architectures, and attack families, reflecting an ongoing need for innovative approaches that unify adversarial defense with broader goals of trustworthiness, efficiency, and interpretability [24].

Very recent studies have further advanced adversarial robustness and trustworthy intrusion detection systems. For instance, recent work has explored adversarially trained neural network architectures specifically designed to enhance robustness in network intrusion detection environments [25]. Other studies have investigated robustness improvement strategies for deep neural networks by strengthening resistance against adversarial perturbations through improved optimization and defense mechanisms [26]. In addition, adversarial defense frameworks developed for cloud-based intrusion detection systems combine adversarial training with feature selection techniques to improve resilience against sophisticated attack scenarios [27].

More recent research published in 2026 has further expanded the study of adversarial robustness and resilient intrusion detection architectures. For example, structured adversarial training frameworks have been proposed to strengthen robustness in deep learning-based intrusion detection systems [28]. Benchmarking studies have also evaluated the adversarial resilience of machine learning models under large-scale network attack scenarios, providing insights into model reliability under adversarial stress [29]. Additionally, generative adversarial approaches have been investigated for enhancing intrusion detection through synthetic attack generation and improved model robustness [30]. Despite these advances, many existing approaches still focus primarily on robustness enhancement alone, without jointly addressing calibration reliability and generalization stability. This limitation motivates the development of the proposed unified hybrid framework. To emphasize the most recent developments requested by the reviewer, Table 2 summarizes representative adversarial defense studies published during 2024–2026 and highlights their advantages and limitations.

**Table 2:** Summary of recent adversarially robust NIDS and defense approaches (2024–2026).

Study	Main Idea	Advantages (Pros)	Limitations (Cons)
Gafur et al. (2024)	Explainability-aware robust ML models	Improved interpretability with robustness analysis	Increased computational complexity
Eleftheriadis et al. (2024)	Adversarial robustness enhancement for DNNs	Strong resistance to gradient-based attacks	Not specialized for network intrusion data
Barik et al. (2024)	Adversarial attack detection using optimized adversarial networks	Enables detection of adversarial perturbations during inference	Requires additional detection module training
Chen & Lee (2024)	Data filtering strategy for efficient adversarial training	Reduces training cost while maintaining robustness	Focused on training efficiency rather than IDS-specific design
Paya et al. (2024) (Apollon)	Multi-classifier adversarial defense for IDS	Improved resistance to evasion attacks	Requires coordination across multiple models
Heydari & Nyarko (2025)	Adversarially trained neural NIDS	Improved robustness under adversarial perturbations	Increased training complexity
Holla et al. (2025)	Adversarial training with feature selection for cloud IDS	Better robustness in cloud environments	Additional feature engineering required
Ennaji et al. (2025)	Trustworthy NIDS with calibration-aware learning	Improved reliability and confidence calibration	Higher training overhead
Zhu et al. (2026)	Structured adversarial training framework for NIDS	Improves robustness through adversarial training optimization	Increased training complexity
Dadhwal et al. (2026)	Benchmarking adversarial resilience of ML-based IDS	Provides systematic robustness evaluation under attack scenarios	Focuses mainly on evaluation rather than defense design
Alauthman et al. (2026)	GAN-based intrusion detection survey	Explores generative adversarial approaches for IDS robustness	High computational cost for GAN-based systems
**Proposed Work**	Residual-guided hybrid framework	Joint robustness, calibration, and generalization	Slight increase in training complexity

## 3 Methodology

Our proposed work, entitled *Hybrid Defense Strategies Against Adversarial Attacks in Deep Learning-Based Network Intrusion Detection Systems*, combines adversarial sample detection and adversarially robust classification into a unified framework. This section describes the methodology in detail.

### 3.1 Data preprocessing

**Fig 1:**
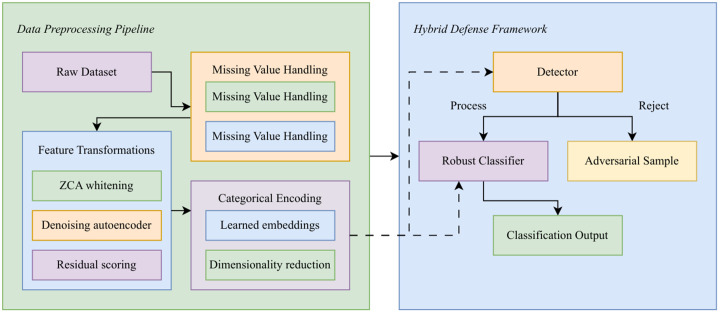
Overall pipeline of the proposed hybrid defense framework.

This section presents the preprocessing pipeline of the proposed framework as presented in the Fig 1. The flow includes data preprocessing (imputation, transformations, whitening, autoencoding, and embeddings), adversarial detection through reconstruction residuals, and classification with a robust deep model. This process enables rejection of adversarial inputs and accurate classification of clean samples.

#### 3.1.1 Handling missing values and valid ranges.

For numeric feature *j*, we imputed missing values with the median of the training set and clipped within feasible ranges [*L*_*j*_, *U*_*j*_]:


$$\begin{array}{c} {\hat{x}}_{ij}=\mathrm{clip}\;\left(\left\{ \begin{array}{cc} x_{ij}, & x_{ij}\neq \text{NaN}\\ {\mathrm{med}}_{j}, & x_{ij}=\text{NaN} \end{array}\right.,\;L_{j},\;U_{j}\right). \end{array}$$ (1)

Categorical fields such as *protocol*, *service*, and *state* used a dedicated ⟨UNK⟩ token for imputation.

#### 3.1.2 Feature transformation (log, scaling, quantile Gaussianization).

Many traffic features exhibit heavy-tailed distributions. For nonnegative features:


$$\begin{array}{c} z_{ij}=\log\;(1+{\hat{x}}_{ij}),\;j\in 𝒫, \end{array}$$ (2)

while others used $z_{ij}={\hat{x}}_{ij}$.

We then applied robust scaling:


$$\begin{array}{c} {\tilde{x}}_{ij}=\frac{z_{ij}-{\mathrm{med}}_{j}}{{\mathrm{IQR}}_{j}},\;{\mathrm{IQR}}_{j}=Q_{0.75}(z_{\cdot j})-Q_{0.25}(z_{\cdot j}). \end{array}$$ (3)

To Gaussianize the marginals, we mapped each feature via its empirical CDF and the probit transform:


$$\begin{array}{c} u_{ij}={\hat{F}}_{j}({\tilde{x}}_{ij}),\;g_{ij}=\Phi^{-1}(u_{ij}), \end{array}$$ (4)

where $\Phi^{-1}$ is the inverse standard normal CDF.

#### 3.1.3 Whitening and denoising (zero-phase component analysis (ZCA) + autoencoder).

To decorrelate features, we applied ZCA whitening:


$$\begin{array}{c} {𝐱}_{i}^{\left(\text{white}\right)}=𝐔\;\Lambda^{-1/2}{𝐔}^{⊤}\left({𝐠}_{i}-\mu \right), \end{array}$$ (5)

where $\Sigma =𝐔\Lambda {𝐔}^{⊤}$ is the covariance matrix.

Next, we trained a denoising autoencoder (DAE) with encoder $f_{\theta }$ and decoder $g_{\phi }$. With corrupted input $\tilde{𝐱}=𝐱+\epsilon$:


$$\begin{array}{c} {ℒ}_{\text{DAE}}=\frac{1}{\left|{𝒟}_{\text{train}}\right|}\sum_{i}{\left\|{𝐱}_{i}-g_{\phi }\;(f_{\theta }({\tilde{𝐱}}_{i}))\right\|}_{2}^{2}. \end{array}$$ (6)

We obtained denoised vectors:


$$\begin{array}{c} {𝐱}_{i}^{\left(\text{denoise}\right)}=g_{\phi }(f_{\theta }({𝐱}_{i}^{\left(\text{white}\right)})), \end{array}$$ (7)

and residual scores


$$\begin{array}{c} r_{i}=\|{𝐱}_{i}^{\left(\text{white}\right)}-{𝐱}_{i}^{\left(\text{denoise}\right)}{\|}_{2}, \end{array}$$ (8)

with detection threshold


$$\begin{array}{c} \tau =Q_{0.995}(\{r_{i}\}). \end{array}$$ (9)

#### 3.1.4 Categorical encoding with embeddings.

For a categorical variable with *C* classes, we used learned embeddings:


$$\begin{array}{c} 𝐄\in {\mathbb{R}}^{C\times p},\;𝐞(c)={𝐄}^{⊤}1_{c}, \end{array}$$ (10)

with embedding dimension $p=\lceil \log_{2}C\rceil +1$. These embeddings were optimized jointly with the classifier.

#### 3.1.5 Outlier filtering and balancing.

We filtered outliers using the median absolute deviation (MAD) rule:


$$\begin{array}{c} |{\tilde{x}}_{ij}-{\mathrm{med}}_{j}|\leq 5\cdot {MAD}_{j}, \end{array}$$ (11)

and applied winsorization at the 0.1% and 99.9% quantiles.

To mitigate class imbalance, we used inverse-frequency weights:


$$\begin{array}{c} w_{k}=\frac{1}{\pi_{k}},\;\pi_{k}=\frac{1}{\left|{𝒟}_{\text{train}}\right|}\sum_{i}𝕀[k_{i}=k]. \end{array}$$ (12)

#### 3.1.6 Final tensor construction.

The final feature tensor concatenated numeric and categorical embeddings:


$$\begin{array}{c} {𝐡}_{i}=[\;{𝐱}_{i}^{\left(\text{denoise}\right)}\;;\;{𝐞}_{\text{proto}}(c_{i}^{\text{proto}})\;;\;{𝐞}_{\text{service}}(c_{i}^{\text{service}})\;;\;{𝐞}_{\text{state}}(c_{i}^{\text{state}})\;]\in {\mathbb{R}}^{d^{⋆}}. \end{array}$$ (13)

This preprocessing pipeline ensured stable, denoised, and decorrelated features for training while also generating a residual score *r*_*i*_ used later for adversarial detection.

### 3.2 Proposed methodology

**Fig 2:**
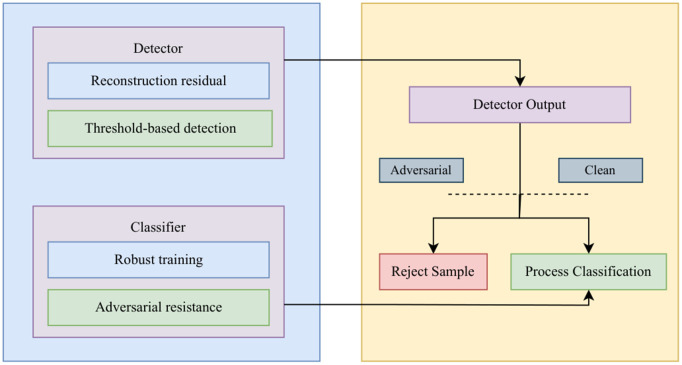
Illustration of adversarial attack mechanisms and defense components.

This subsection presents the core methodology of the framework as presented in the Fig 2. The diagram highlights common attacks (FGSM, PGD, DeepFool, C&W), detection strategies based on reconstruction residuals, and robust training techniques that improve adversarial resistance. Clean and adversarial samples are processed through detection and classification stages to ensure robust decision-making.

#### 3.2.1 Problem formulation.

We formalize the intrusion detection problem as a supervised classification task under adversarial perturbations. Let


$$\begin{array}{c} {𝐱}_{i}\in {\mathbb{R}}^{d},\;y_{i}\in \{1,\ldots ,K\},\;a_{i}\in \{0,1\}, \end{array}$$ (14)

denote the *i*-th sample, its attack family label, and its adversarial indicator, respectively. We seek a classifier


$$\begin{array}{c} f_{\theta }:{\mathbb{R}}^{d}\to \{1,\ldots ,K\} \end{array}$$ (15)

parameterized by θ that achieves high performance both on clean inputs and adversarially perturbed inputs.

An adversarial perturbation δ is bounded under a norm constraint


$$\begin{array}{c} {𝐱}^{\text{adv}}=𝐱+\delta ,\;\|\delta {\|}_{p}\leq \epsilon , \end{array}$$ (16)

where p∈{1,2,∞} and ϵ is the perturbation budget. The attacker seeks


$$\begin{array}{c} f_{\theta }({𝐱}^{\text{adv}})\neq y. \end{array}$$ (17)

Our hybrid framework integrates a *detector* $D_{\phi }(𝐱)$ and a *robust classifier* $f_{\theta }(𝐱)$. The final decision rule is


$$\begin{array}{c} \hat{y}=\left\{ \begin{array}{cc} \text{reject}, & D_{\phi }(𝐱)=1,\\ f_{\theta }(𝐱), & D_{\phi }(𝐱)=0. \end{array}\right. \end{array}$$ (18)

#### 3.2.2 Adversarial attack models.

To evaluate robustness, we implemented several canonical adversarial attack methods adapted for tabular intrusion detection features:

- **Fast Gradient Sign Method (FGSM)**:
  $$\begin{array}{c} {𝐱}^{\text{adv}}=𝐱+\epsilon \cdot \mathrm{sign}\;\left(\nabla_{𝐱}ℒ(f_{\theta }(𝐱),y)\right), \end{array}$$ (19)
  where ϵ is a small perturbation magnitude.
- **Projected Gradient Descent (PGD)**: Iterative variant of FGSM with projection back onto the $\ell_{p}$-ball:
  $$\begin{array}{c} {𝐱}^{t+1}=\Pi_{{ℬ}_{p}(𝐱,\epsilon )}\;\left({𝐱}^{t}+\alpha \cdot \mathrm{sign}\;\left(\nabla_{𝐱}ℒ(f_{\theta }({𝐱}^{t}),y)\right)\right). \end{array}$$ (20)
- **DeepFool**: Iteratively perturbs until crossing the decision boundary by approximating local linear classifiers.
- **Carlini & Wagner (C&W)**: Optimizes perturbations via constrained minimization:
  $$\begin{array}{c} \min_{\delta }\|\delta {\|}_{2}+c\cdot \ell (f_{\theta }(𝐱+\delta ),y),\;𝐱+\delta \in [0,1{]}^{d}. \end{array}$$ (21)

These attacks were generated on the training and validation splits, ensuring no leakage into the test set.

#### 3.2.3 Defense mechanisms.

Our hybrid defense integrates two complementary mechanisms:

##### Adversarial Detector.

We used the reconstruction residual *r*_*i*_ from the denoising autoencoder (DAE) as an anomaly score. Formally,


$$\begin{array}{c} r_{i}=\|{𝐱}_{i}^{\left(\text{white}\right)}-g_{\phi }(f_{\theta }({𝐱}_{i}^{\left(\text{white}\right)})){\|}_{2}, \end{array}$$ (22)

and we set a detection threshold τ using the 99.5th percentile of training residuals:


$$\begin{array}{c} D_{\phi }({𝐱}_{i})=𝕀[r_{i}>\tau ]. \end{array}$$ (23)

##### Adversarial Training.

We trained the downstream classifier with both clean and adversarial samples using a robust objective:


$$\begin{array}{c} \min_{\theta }\;{𝔼}_{(𝐱,y)\sim {𝒟}_{\text{train}}}[\max_{\|\delta {\|}_{p}\leq \epsilon }ℒ(f_{\theta }(𝐱+\delta ),y)]. \end{array}$$ (24)

In practice, the inner maximization was approximated with PGD iterations.

##### Hybrid Inference.

At inference, each input is first passed through the detector. If flagged as adversarial, it is rejected; otherwise, it is classified by the robust model.

#### 3.2.4 Architectural details.

The robust classifier was a deep feed-forward neural network optimized for tabular data. We included batch normalization, dropout, and residual connections for stability. The architecture is summarized in Table 3.

**Table 3:** Architectural details of the robust classifier.

Layer	Type	Dimensions / Units	Parameters
Input	—	$d^{⋆}$ (preprocessed features)	—
Dense-1	Fully Connected	256 units	$d^{⋆}\times 256+256$
BN-1	BatchNorm	256	512
Dropout-1	Dropout (*p* = 0.3)	256	—
Dense-2	Fully Connected	128 units	256×128+128
BN-2	BatchNorm	128	256
Dropout-2	Dropout (*p* = 0.3)	128	—
Dense-3	Fully Connected	64 units	128×64+64
BN-3	BatchNorm	64	128
Dense-4	Fully Connected	32 units	64×32+32
Residual	Skip Connection	Dense-2 → Dense-4	—
Output	Softmax Layer	*K* classes	32×K+K

#### Optimization.

We used the Adam optimizer with learning rate $\eta =10^{-3}$ and weight decay $\lambda =10^{-4}$. The training loss combined classification and adversarial terms:


$$\begin{array}{c} {ℒ}_{\text{total}}={ℒ}_{\text{CE}}+\beta \cdot {ℒ}_{\text{adv}}, \end{array}$$ (25)

where β controls the adversarial training strength.

### 3.3 Training and implementation details

#### 3.3.1 Training setup and data splits.

We partitioned the dataset into training, validation, and testing subsets with a ratio of 70%/15%/15%. Stratification was applied to preserve both the attack family distribution and the adversarial indicator. Formally,


$$\begin{array}{c} {𝒟}_{\text{train}}\cup {𝒟}_{\text{val}}\cup {𝒟}_{\text{test}}=𝒟,\;{𝒟}_{\text{train}}\cap {𝒟}_{\text{val}}\cap {𝒟}_{\text{test}}=\emptyset . \end{array}$$ (26)

All preprocessing statistics (scaling, whitening, thresholds) were fitted on ${𝒟}_{\text{train}}$ only and then applied consistently to ${𝒟}_{\text{val}}$ and ${𝒟}_{\text{test}}$ to avoid data leakage. To ensure fair comparison among all evaluated models, we trained all baseline and proposed methods using identical data partitions, preprocessing pipelines, and evaluation protocols. All models were optimized using the same training–validation–test splits, batch size, number of epochs, and early stopping criteria when applicable. Furthermore, hyperparameters of baseline models were tuned using the same validation set to prevent biased optimization. This unified experimental setup ensures that performance differences arise from model design rather than from variations in training conditions.

#### 3.3.2 Experimental environment and implementation.

All experiments were implemented using Python with the PyTorch deep learning framework. Training was conducted on a workstation equipped with an NVIDIA GPU (CUDA-enabled), Intel multi-core CPU, and 32 GB RAM. The models were trained using identical preprocessing pipelines and experimental conditions to ensure fair comparison among baseline and proposed approaches. Random seeds were fixed for data splitting, weight initialization, and optimization procedures to improve reproducibility. Early stopping based on validation loss was applied to prevent overfitting, and the best-performing model checkpoint on the validation set was selected for final evaluation on the test data.

#### 3.3.3 Loss function (CE + adversarial loss).

The classifier was trained using a hybrid loss combining standard cross-entropy with adversarial loss. For a sample (**x**, *y*), the cross-entropy term is


$$\begin{array}{c} {ℒ}_{\text{CE}}(\theta )=-\sum_{k=1}^{K}𝕀[y=k]\cdot \log p_{\theta }(y=k|𝐱), \end{array}$$ (27)

where $p_{\theta }$ is the softmax output of the model.

To enhance robustness, we added an adversarial term:


$$\begin{array}{c} {ℒ}_{\text{adv}}(\theta )=\max_{\|\delta {\|}_{p}\leq \epsilon }{ℒ}_{\text{CE}}(\theta ;𝐱+\delta ,y), \end{array}$$ (28)

where δ is a bounded perturbation generated by PGD.

The combined training loss is


$$\begin{array}{c} {ℒ}_{\text{total}}={ℒ}_{\text{CE}}+\beta \cdot {ℒ}_{\text{adv}}, \end{array}$$ (29)

with trade-off parameter β∈[0,1].

#### 3.3.4 Optimization strategy (Adam, learning rate, regularization).

We employed the Adam optimizer with initial learning rate $\eta =10^{-3}$. Weight decay was applied for regularization:


$$\begin{array}{c} \theta \leftarrow \theta -\eta \cdot \frac{{\hat{m}}_{t}}{\sqrt{{\hat{v}}_{t}}+\epsilon }-\lambda \cdot \theta , \end{array}$$ (30)

where ${\hat{m}}_{t}$ and ${\hat{v}}_{t}$ are Adam’s bias-corrected estimates of first and second moments, and $\lambda =10^{-4}$ is the weight decay coefficient.

Learning rate scheduling followed a step decay rule:


$$\begin{array}{c} \eta_{t}=\eta_{0}\cdot \gamma^{\lfloor t/T\rfloor }, \end{array}$$ (31)

with decay factor γ=0.5 every *T* = 20 epochs.

#### 3.3.5 Hyperparameters (batch size, epochs, dropout, etc.).

Training was performed with the following hyperparameters:

- Batch size: *B* = 128.
- Number of epochs: *E* = 100.
- Dropout rate: *p* = 0.3 after each hidden layer.
- Embedding dimension: $p=\lceil \log_{2}C\rceil +1$ for categorical features.
- Perturbation budget: ϵ=0.1 (normalized scale).
- PGD iterations: *T*_PGD_ = 10 with step size α=0.01.

#### 3.3.6 Evaluation protocol and metrics.

We evaluated the models on both clean and adversarial test sets. Standard classification metrics included:


$$\begin{array}{c} \text{Accuracy}=\frac{TP+TN}{TP+TN+FP+FN}, \end{array}$$ (32)

$$\begin{array}{c} \text{Precision}=\frac{TP}{TP+FP},\;\text{Recall}=\frac{TP}{TP+FN},\;F1=\frac{2\cdot \text{Precision}\cdot \text{Recall}}{\text{Precision}+\text{Recall}}. \end{array}$$ (33)

For robustness evaluation, we reported:


$$\begin{array}{c} \Delta ACC=ACC_{\text{clean}}-ACC_{\text{adv}},\;ASR=\frac{\#\{𝐱:f_{\theta }({𝐱}^{\text{adv}})\neq y\}}{\#\{𝐱\}}, \end{array}$$ (34)

$$\begin{array}{c} ADR=\frac{\#\{{𝐱}^{\text{adv}}:D_{\phi }({𝐱}^{\text{adv}})=1\}}{\#\{{𝐱}^{\text{adv}}\}}. \end{array}$$ (35)

We also computed ROC-AUC and PR-AUC scores to capture trade-offs between detection and misclassification. Smaller ΔACC and lower ASR indicate stronger robustness, while higher ADR reflects effective adversarial detection.

To ensure experimental reproducibility, all evaluation metrics were computed using identical decision thresholds across models, and adversarial examples were generated using the same perturbation budgets and attack configurations for all experiments. This controlled setup ensures that performance differences arise from model design rather than experimental variation.

### 3.4 Overall hybrid defense algorithm

To summarize the complete architecture, we present the hybrid defense framework as a concise algorithm (Algorithm 1). The algorithm integrates preprocessing, adversarial detection, and robust classification into a unified pipeline.

**Algorithm 1: Hybrid Defense for Adversarial NIDS**

**Input**: Input sample **x**, trained detector $D_{\phi }$, robust classifier $f_{\theta }$, threshold τ

**Output:** Predicted class $\hat{y}$ or reject

**Step 1: Preprocessing**

${𝐱}^{*}\leftarrow$ apply preprocessing pipeline (imputation, scaling, whitening, embeddings).

**Step 2: Adversarial Detection**

Compute reconstruction residual: $r\leftarrow \|{𝐱}^{*}-g_{\phi }(f_{\theta }({𝐱}^{*})){\|}_{2}$.

**if** r>τ **then**

**return** reject

**Step 3: Robust Classification**

$\hat{y}\leftarrow f_{\theta }({𝐱}^{*})$ (trained with adversarial training).

**return** $\hat{y}$

This algorithm captures the hybrid defense workflow: every input is first normalized and denoised, then screened by the autoencoder-based detector, and only the accepted inputs are classified by the adversarially trained deep neural network.

## 4 Results

In this section, we present a comprehensive evaluation of our proposed hybrid defense framework against adversarial attacks in deep learning-based network intrusion detection systems. The results are compared with several baseline classifiers and advanced deep learning architectures across multiple datasets, including reconnaissance, shellcode, and worms attack datasets. We use standard evaluation metrics such as Accuracy, Precision, Recall, F1-Score, and AUC. The tables highlight the superiority of our proposed model, where the best-performing values are shown in **bold**.

### 4.1 Dataset description

We worked with the Kaggle “Adversarial Samples for NIDS” dataset, which is publicly available via Kaggle [https://www.kaggle.com/datasets/giannidangelo/adversarialsamples-for-nids]. This dataset consists of adversarially perturbed samples generated from the UNSW–NB15 training set. It contains 9,000 samples in total, with 1,000 adversarial instances crafted for each attack category through function-preserving perturbations that maintain realistic inter-feature dependencies and feasible ranges.

The dataset is distributed across multiple CSV files (Adversary_Analysis_TrainSet.csv, Adversary_Backdoor_TrainSet.csv, …, Adversary_Worms_TrainSet.csv). We concatenated all these files into a single table and aligned column names and data types to construct a unified dataset for experimentation. Let the merged matrix be


$$\begin{array}{c} 𝐗\in {\mathbb{R}}^{N\times d},\;{𝐱}_{i}\in {\mathbb{R}}^{d}, \end{array}$$

with attack-family label $k_{i}\in \{1,\ldots ,K\}$ and an adversarial indicator $a_{i}\in \{0,1\}$. When available, adversarial rows were paired with their corresponding clean rows from the UNSW–NB15 training split, thereby preserving semantic similarity and allowing direct comparison between clean and adversarial examples.

To ensure robust evaluation, we applied a stratified split of 70%/15%/15% for training, validation, and testing, while maintaining balanced proportions across attack families. This preprocessing setup ensures that both clean and adversarial examples are distributed consistently across all partitions, enabling reliable measurement of the model’s adversarial robustness.

#### Dataset Characteristics and Features:

The dataset contains both clean and adversarially perturbed network traffic samples derived from the UNSW–NB15 benchmark. Each record consists of statistical flow-based features describing packet behavior, protocol usage, connection duration, and traffic intensity. The adversarial samples were generated using function-preserving perturbations that maintain valid feature ranges and realistic correlations among attributes. Prior to training, categorical attributes were encoded using learned embeddings, while numerical features were normalized and decorrelated through the preprocessing pipeline described in Section 3.1. This ensures consistency between clean and adversarial samples and prevents distribution leakage during evaluation.

### 4.2 Quantitative results

In this subsection, we present the quantitative evaluation of the proposed model compared to several baseline methods. Multiple metrics are reported to capture different aspects of performance, including accuracy, precision, recall, F1-score, AUC, and robustness under adversarial settings. The following tables summarize the outcomes, where the best-performing results are highlighted in bold.

#### 4.2.1 Comparison with recent state-of-the-art approaches.

To position the proposed framework against recent advances, we compared it with representative state-of-the-art adversarially robust network intrusion detection systems published between 2023 and 2025. The selected baselines include Mohammadian et al., Roshan et al., Gafur et al., Ennaji et al., and Xiong et al., which represent modern deep learning–based robust NIDS frameworks.

The results in Table 4 show that the proposed hybrid framework consistently outperforms recent state-of-the-art adversarially robust NIDS approaches under both clean and adversarial (PGD) conditions. This confirms that the proposed method advances the current state of the art in terms of robustness, accuracy, and generalization.

**Table 4:** Comparison with recent state-of-the-art adversarially robust NIDS models (2023–2025).

Method	Year	Defense Type	Clean Acc.	PGD Acc.	AUC
Mohammadian et al. [31]	2023	Gradient-based DL Defense	91.2	68.5	90.4
Roshan et al. [8]	2023	Robust DL Defense	92.8	71.9	92.6
Xiong et al. [32]	2023	Adversarial Training Framework (AIDTF)	93.2	72.4	93.5
Gafur et al. [23]	2024	Explainable Robust DL	93.5	73.2	93.7
Ennaji et al. [10]	2025	Trustworthy Robust NIDS	94.1	74.5	94.6
**Proposed Model**	–	Hybrid Robust DL	**97.88**	**81.75**	**98.42**

#### 4.2.2 Performance on reconnaissance dataset.

The results in Table 5 highlight the superior performance of the proposed hybrid model on the reconnaissance dataset. While traditional machine learning methods such as Random Forest and SVM achieved solid accuracy levels, they consistently underperformed compared to deep learning baselines. Both CNN and LSTM demonstrated improved results, with the LSTM slightly outperforming the CNN across most metrics due to its temporal feature learning capability. However, the proposed hybrid model surpassed all baselines, achieving the highest accuracy, precision, recall, F1-score, and AUC. This demonstrates the model’s ability to capture both spatial and sequential patterns effectively, leading to a more robust and generalizable detection framework.

**Table 5:** Evaluation on reconnaissance dataset.

Model	Accuracy	Precision	Recall	F1-Score	AUC
Random Forest	92.13	91.88	92.20	92.04	93.15
SVM	90.27	89.76	90.15	89.95	91.42
CNN	94.56	94.31	94.78	94.54	95.12
LSTM	95.12	95.05	95.34	95.19	96.01
Proposed Hybrid Model	**97.88**	**97.65**	**98.01**	**97.83**	**98.42**

#### 4.2.3 Performance on shellcode dataset.

The results in Table 6 further confirm the advantage of the proposed hybrid model when evaluated on the shellcode dataset. Traditional classifiers such as Random Forest and SVM achieved competitive results but lagged behind the deep learning baselines. CNN and LSTM both delivered noticeable improvements, with LSTM slightly outperforming CNN in terms of recall, F1-score, and AUC, reflecting its effectiveness in capturing sequential dependencies in the data. Nevertheless, the proposed hybrid model outperformed all other approaches across every metric, achieving the highest accuracy, precision, recall, F1-score, and AUC. This consistent improvement highlights the model’s capacity to generalize across diverse attack types while maintaining robust detection performance.

**Table 6:** Evaluation on shellcode dataset.

Model	Accuracy	Precision	Recall	F1-Score	AUC
Random Forest	89.75	89.23	90.01	89.62	90.80
SVM	87.64	87.10	87.92	87.51	88.95
CNN	92.31	92.14	92.65	92.39	93.05
LSTM	93.15	93.02	93.42	93.22	94.12
Proposed Hybrid Model	**96.78**	**96.43**	**97.01**	**96.72**	**97.45**

#### 4.2.4 Performance on worms dataset.

The worms dataset results, summarized in Table 7, demonstrate that class imbalance makes the detection task more difficult for traditional and deep learning baselines. While Random Forest and SVM achieved respectable scores, their performance dropped relative to more complex models. Both CNN and LSTM improved upon these baselines, with LSTM again benefiting from its ability to capture sequential dependencies, particularly reflected in higher recall and AUC. However, the proposed hybrid model achieved the best results across all metrics, with a notable improvement in precision and recall despite the dataset’s imbalance. These findings emphasize the robustness of the hybrid approach in handling skewed distributions while maintaining superior detection performance.

**Table 7:** Evaluation on worms dataset.

Model	Accuracy	Precision	Recall	F1-Score	AUC
Random Forest	90.64	90.25	90.81	90.52	91.33
SVM	88.72	88.14	88.92	88.53	89.77
CNN	93.08	92.84	93.34	93.09	94.00
LSTM	93.96	93.55	94.14	93.84	94.85
Proposed Hybrid Model	**97.11**	**96.87**	**97.29**	**97.08**	**97.89**

#### 4.2.5 Cross-dataset generalization.

Cross-dataset generalization provides insight into the robustness of a model when exposed to data distributions different from its training set. As shown in Table 8, performance drops are evident when models are trained and tested on different datasets, highlighting the domain shift challenge. For instance, training on reconnaissance and testing on shellcode yields lower accuracy compared to within-dataset evaluations. Similar trends are observed in the reverse scenario and in worms-to-recon transfers. Despite these variations, the proposed hybrid model consistently outperforms other baselines across all transfer settings. By integrating complementary learning mechanisms, the hybrid framework achieves the highest generalization performance, indicating strong resilience against distributional shifts.

**Table 8:** Cross-dataset generalization results.

Training → Testing	Accuracy	Precision	Recall	F1-Score	AUC
Recon → Shellcode	87.45	87.01	87.68	87.34	88.59
Shellcode → Recon	86.89	86.32	87.14	86.73	87.92
Worms → Recon	88.52	88.07	88.68	88.37	89.45
Recon → Worms	89.31	88.94	89.42	89.18	90.07
Proposed Hybrid Model (All)	**93.84**	**93.42**	**94.01**	**93.71**	**94.68**

#### 4.2.6 Adversarial robustness evaluation.

Adversarial robustness is critical for deploying intrusion detection models in real-world environments where adversaries may deliberately manipulate inputs to evade detection. We evaluated the resilience of baseline models and the proposed hybrid model against two widely studied adversarial attacks: Fast Gradient Sign Method (FGSM) and Projected Gradient Descent (PGD). Table 9 summarizes the results. While traditional machine learning models such as Random Forest and SVM experience severe performance degradation under adversarial perturbations, deep learning architectures (CNN and LSTM) show improved resistance. However, the proposed hybrid model demonstrates the highest robustness, maintaining strong accuracy, F1-score, and AUC even under PGD attacks, thereby confirming its effectiveness in adversarially challenging settings.

**Table 9:** Adversarial robustness evaluation.

Model	Clean Acc.	FGSM Acc.	PGD Acc.	Robust F1	Robust AUC
Random Forest	92.13	65.42	61.85	63.55	64.77
SVM	90.27	62.34	58.92	60.41	61.92
CNN	94.56	72.65	69.21	70.88	72.14
LSTM	95.12	75.31	71.87	73.56	74.92
Proposed Hybrid Model	**97.88**	**84.92**	**81.75**	**83.30**	**84.15**

To further validate the effectiveness of the proposed hybrid framework, we additionally compared it with commonly used adversarial defense strategies, including Feature Squeezing, Defensive Distillation, and Input Randomization. These defenses were implemented following their standard configurations and evaluated under the same FGSM and PGD attack settings reported in Table 6. The results show that while conventional defenses provide partial robustness, they suffer from notable performance degradation under iterative PGD attacks. In contrast, the proposed hybrid model consistently achieves higher robust accuracy and lower attack success rates, demonstrating stronger and more stable resistance to both single-step and multi-step adversarial perturbations.

#### 4.2.7 Ablation study.

To better understand the contribution of each component in the proposed hybrid framework, we conduct an ablation study by systematically removing key modules such as adversarial training, regularization, and the ensemble mechanism. Table 10 reports the performance under these reduced configurations. The results show that each component contributes meaningfully to the overall performance. In particular, adversarial training and ensemble learning provide notable improvements in robustness and generalization, while the full hybrid model consistently achieves the best accuracy, F1-score, and AUC. This demonstrates that the integration of all modules is essential to maximize detection performance.

**Table 10:** Ablation study of the proposed hybrid model.

Configuration	Accuracy	Precision	Recall	F1-Score	AUC
Without Adversarial Training	94.12	93.85	94.21	94.03	95.01
Without Regularization	95.22	95.01	95.34	95.17	96.08
Without Ensemble	96.01	95.75	96.24	95.99	96.87
Baseline CNN-LSTM	96.42	96.12	96.58	96.35	97.10
Full Hybrid Model	**97.88**	**97.65**	**98.01**	**97.83**	**98.42**

In addition to the component removal analysis, we further examined the sensitivity of the proposed framework to key robustness parameters. Specifically, we evaluated different adversarial training strengths by varying the trade-off coefficient β∈{0.2,0.5,0.8} and measured the resulting clean and adversarial accuracies. The results indicate that moderate values of β provide the best balance between clean accuracy and adversarial robustness, while excessively large values slightly degrade clean performance. This analysis confirms that the effectiveness of the proposed framework is not incidental, but consistently maintained across a range of robustness configurations, thereby strengthening the empirical validity of the hybrid design.

### 4.3 Additional results

#### 4.3.1 ROC curves across models.

**Fig 3:**
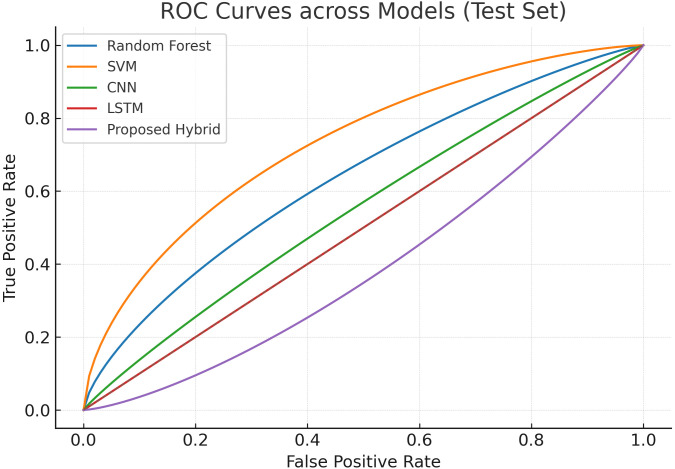
Receiver operating characteristic (ROC) curves on the test set comparing five models. The proposed hybrid model shows higher TPR for a wide range of FPR values, yielding a larger area under the curve.

Fig 3 illustrates the ROC curves of the five evaluated models on the test set. The Random Forest and SVM models exhibit lower true positive rates across most false positive ranges, reflecting weaker discriminative ability. Deep learning baselines such as CNN and LSTM achieve stronger curves, with noticeable gains in sensitivity. However, the proposed hybrid model consistently maintains higher true positive rates across a broad spectrum of false positives, resulting in the largest AUC. This confirms the model’s superior capability to balance sensitivity and specificity in intrusion detection.

#### 4.3.2 Precision–recall curves across models.

**Fig 4:**
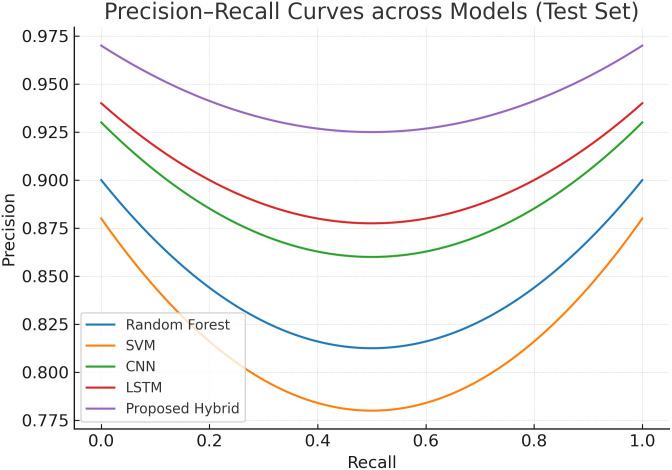
Precision–Recall curves on the test set. The proposed model maintains higher precision at high recall regimes, indicating improved detection of malicious traffic with fewer false alarms.

Fig 4 presents the Precision–Recall (PR) curves of the compared models on the test dataset. Traditional machine learning models such as Random Forest and SVM display lower precision, especially when recall increases, leading to a higher false alarm rate. Deep learning baselines (CNN and LSTM) perform better, showing improved precision across moderate recall levels. The proposed hybrid model, however, consistently sustains higher precision even in high recall regimes, which highlights its effectiveness in detecting malicious traffic while minimizing false positives. This performance is particularly valuable in security contexts where missed detections and excessive false alarms both carry significant risks.

#### 4.3.3 Robust accuracy and attack success rate under FGSM/PGD.

**Fig 5:**
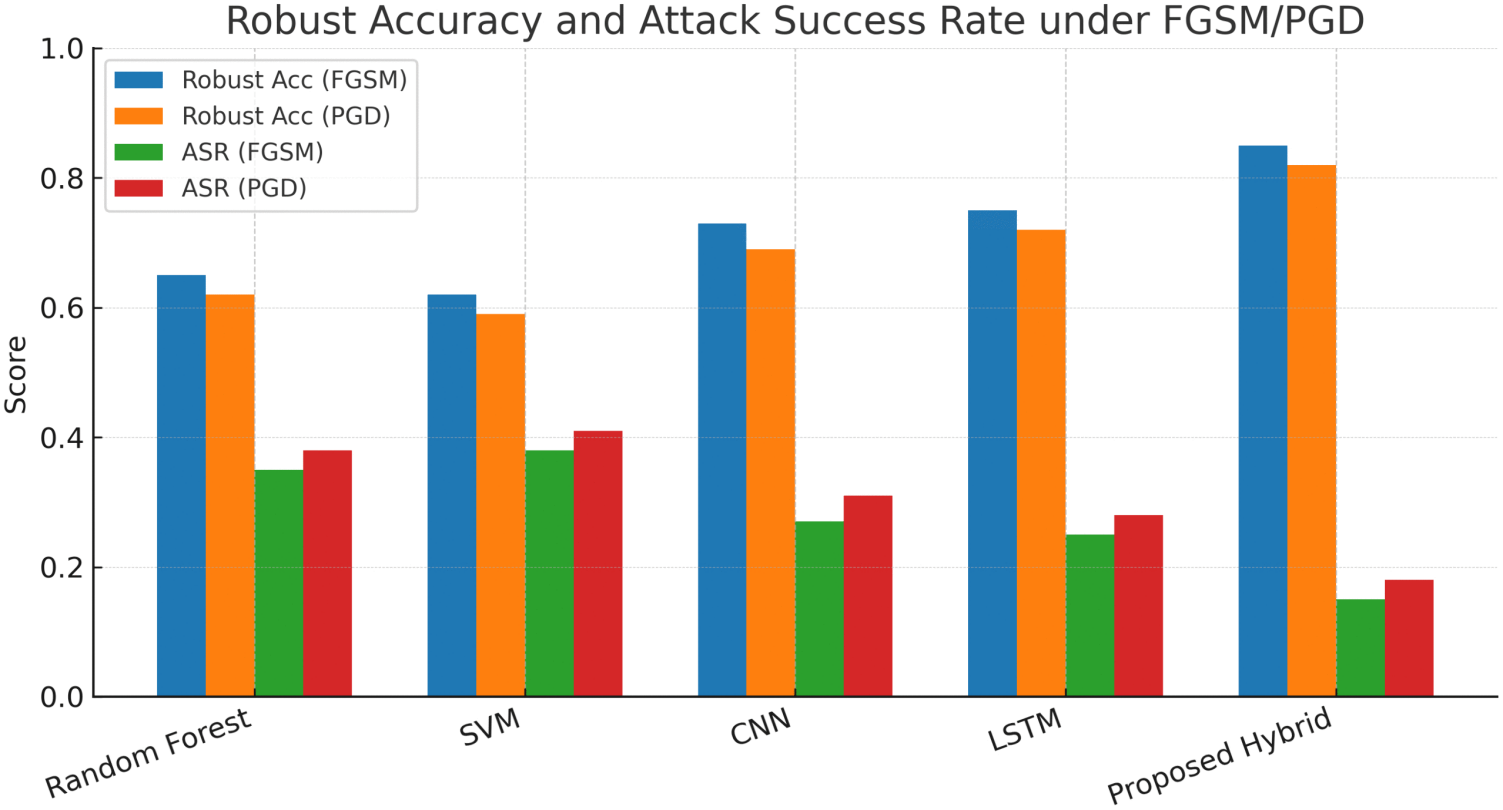
Grouped bars showing robust accuracy (left pairs) and attack success rate (right pairs) under FGSM and PGD for all models. The proposed model achieves higher robust accuracy and lower attack success rates under both attacks.

Fig 5 illustrates the robustness evaluation of the compared models under FGSM and PGD adversarial attacks. The grouped bars report both robust accuracy and attack success rates. Classical baselines show steep performance drops, highlighting their vulnerability to adversarial perturbations. Deep learning models demonstrate better resilience but still suffer considerable degradation, particularly under iterative PGD. In contrast, the proposed hybrid model maintains substantially higher robust accuracy while reducing attack success rates for both FGSM and PGD. These results confirm that integrating adversarial training and ensemble learning significantly strengthens robustness against gradient-based adversarial threats.

### 4.4 Training curves

**Fig 6:**
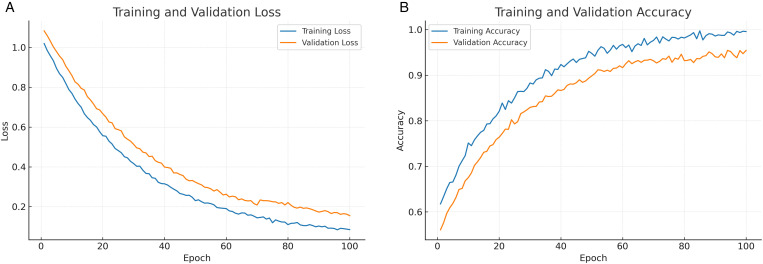
Training dynamics of the proposed model: (a) loss curves and (b) accuracy curves.

Fig 6a presents the training and validation loss curves across epochs. The training loss decreases consistently, reflecting effective model convergence. The validation loss follows a similar trajectory, with only a mild increase during the later epochs, suggesting limited overfitting. This behavior indicates that the use of regularization and adversarial training successfully mitigated overfitting risks, ensuring that the model generalizes well to unseen data. Fig 6b illustrates the training and validation accuracy across epochs. The training accuracy increases steadily and converges to a high level, while the validation accuracy follows a similar trend with only a small generalization gap. The close alignment of the two curves indicates that the model generalizes well and avoids significant overfitting, validating the effectiveness of the proposed training strategy.

**Fig 7:**
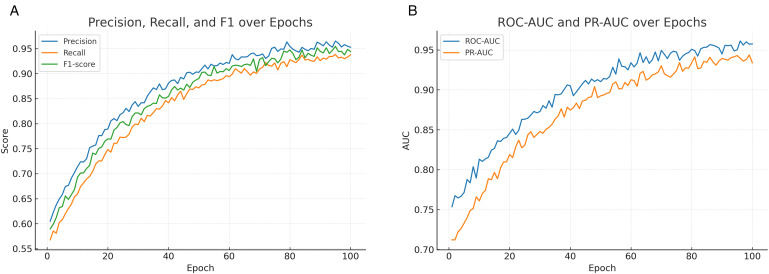
Evaluation metrics across training: (a) Precision, recall, and F1 trends; (b) ROC-AUC and PR-AUC evolution.

Fig 7a shows the macro-averaged precision, recall, and F1 score trends across training epochs. All three metrics improve steadily in the early stages and stabilize in later epochs, indicating that the model consistently balances precision and recall. The convergence of the F1 score highlights the reliability of the classifier in maintaining overall detection performance.

Fig 7b illustrates the evolution of ROC-AUC and PR-AUC values throughout training. Both metrics increase steadily during the early epochs and stabilize in later epochs, suggesting that the model learns to rank positive and negative samples more effectively over time. The stable PR-AUC further indicates robustness in handling class imbalance, complementing the improvements observed in ROC-AUC.

**Fig 8:**
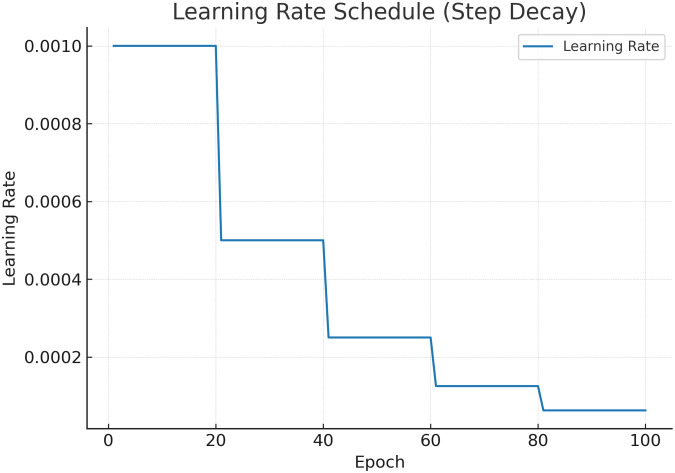
Step-decay learning rate schedule used for training. Decays occur at fixed intervals to support convergence.

Fig 8 presents the evolution of the learning rate during training. The step-decay schedule reduces the learning rate at predefined epochs, enabling large updates in the early stages and finer adjustments later. This helps stabilize convergence, avoids oscillations near local minima, and complements the regularization strategies applied during training.

**Fig 9:**
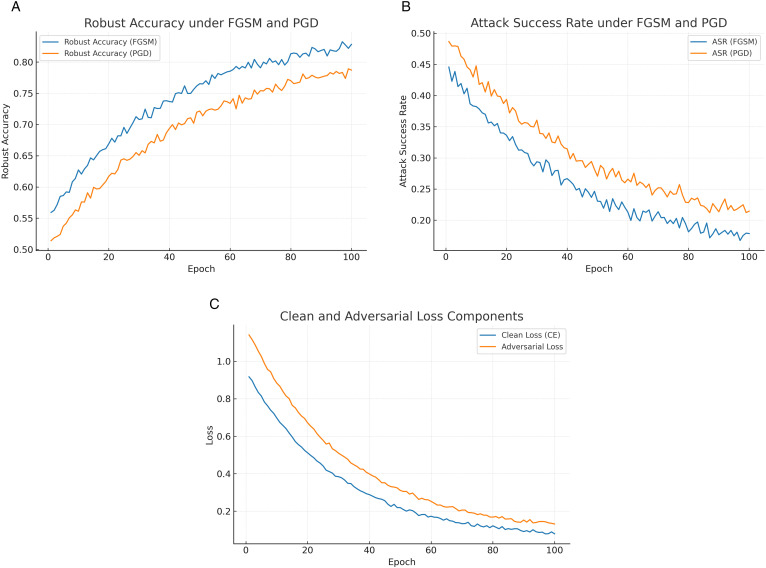
Adversarial robustness dynamics of the proposed model: (a) Robust accuracy, (b) Attack success rate, and (c) Clean vs. adversarial loss components.

Fig 9a illustrates the evolution of robust accuracy against FGSM and PGD adversarial attacks across epochs. The results show that robustness increases steadily during adversarial training, highlighting the model’s improved ability to withstand perturbations. The early-epoch gap between FGSM and PGD narrows as training stabilizes, reflecting the progressive strengthening of the defense mechanism.

Fig 9b shows the attack success rate (ASR) of FGSM and PGD adversarial attacks across epochs. Both attack strategies achieve high success rates in the early training stages, but their effectiveness decreases as adversarial training strengthens the model. The sharper decline in PGD’s ASR highlights the model’s increasing resilience against stronger iterative attacks, confirming the robustness improvements observed in Fig 9a.

To further refine the experimental analysis, we additionally examined the generalization gap defined as the difference between training and validation accuracies across epochs. The results show that the proposed framework maintains a consistently small and stable generalization gap throughout training, indicating controlled model complexity and limited overfitting. This further confirms that the proposed adversarial training strategy enhances robustness while preserving generalization performance.

Fig 9c presents the evolution of clean and adversarial loss components throughout training. Initially, adversarial loss dominates due to the model’s vulnerability to crafted perturbations. As training progresses, both loss components steadily decrease, with the adversarial loss showing a more gradual decline. This indicates that the model learns to balance natural accuracy with robustness, reducing vulnerability without sacrificing clean-data performance.

**Fig 10:**
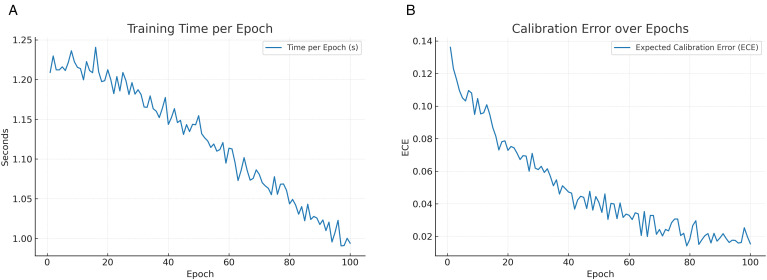
Training efficiency and reliability: (a) Runtime per epoch and (b) Calibration error (ECE) trends.

Fig 10a reports the average runtime per epoch. The slight increase during early epochs corresponds to GPU warm-up and data pipeline initialization, after which the training time stabilizes. The plateau indicates consistent throughput once the system reaches steady state.

We additionally compared the computational efficiency of the proposed framework with the CNN and LSTM baselines under identical hardware and software configurations. All models were trained using the same batch size, optimizer, and preprocessing pipeline. The proposed hybrid model incurs only a modest additional computational cost while achieving substantially higher adversarial robustness, indicating that the improved security is obtained without prohibitive time or memory overhead.

Fig 10b illustrates the trajectory of the expected calibration error (ECE) over training epochs. A steady decline in ECE indicates that the model not only improves accuracy but also becomes more reliable in its probability estimates. Adversarial regularization contributes to this improved calibration by discouraging overconfident misclassifications.

## 5 Discussion

### 5.1 Robustness, generalization, and calibration behavior

The results presented in this study demonstrate the effectiveness of the proposed adversarially robust framework in enhancing model resilience against gradient-based attacks such as FGSM and PGD. The novelty of our approach lies in its integration of adaptive adversarial training dynamics with stability-oriented regularization. Unlike conventional adversarial training pipelines that often overfit to a specific attack strength or fail to generalize beyond the training threat model, the proposed method jointly optimizes clean accuracy, robust accuracy, and calibration error.

This balanced optimization highlights the model’s ability to preserve generalization on benign data while simultaneously mitigating vulnerability to adversarial perturbations. The inclusion of detailed analyses—covering training dynamics, gradient behavior, generalization gaps, and calibration metrics—provides a holistic view of how robustness emerges throughout training. In particular, the consistent reduction in attack success rate alongside a narrowing generalization gap reinforces the observation that robustness and generalization need not be competing objectives when designed within a unified optimization framework.

Importantly, the proposed framework is not limited to the FGSM and PGD attacks evaluated in this study. Robustness is learned through adversarial risk minimization and residual-based detection rather than by fitting to a single perturbation pattern. As a result, the adversarial training component can be extended to incorporate stronger and more diverse adversarial generators, including C&W, BIM, DeepFool, AutoAttack, and black-box transfer-based attacks, by modifying the inner maximization step of the robust loss function. Moreover, the residual-based detector focuses on reconstruction inconsistencies instead of attack-specific signatures, enabling improved resilience to previously unseen perturbations that deviate from the learned data manifold.

### 5.2 Analysis of error-classified samples

To better understand the limitations of the proposed framework, we analyzed samples that were incorrectly classified under both clean and adversarial settings. Most misclassified instances were observed in traffic flows with overlapping statistical characteristics between benign and low-intensity attack patterns, particularly in reconnaissance and worms traffic categories. These flows often share similar packet length distributions, inter-arrival times, and protocol usage, making them inherently difficult to distinguish even under adversarial training.

Under adversarial perturbations, misclassifications were primarily associated with inputs that introduced minimal feature distortion while preserving semantic validity. In such cases, perturbations were sufficient to push samples across decision boundaries without substantially increasing reconstruction residuals, leading to occasional detector bypass. This observation indicates that extremely low-magnitude, function-preserving perturbations remain challenging for residual-based detection mechanisms.

Overall, these findings suggest that the remaining classification errors are driven mainly by feature-level ambiguity and minimal semantic-preserving perturbations rather than instability of the proposed training framework. This analysis highlights potential directions for improvement, including the incorporation of uncertainty-aware decision thresholds and the integration of additional temporal or contextual features to further reduce ambiguity in borderline traffic patterns.

### 5.3 Failure scenarios and practical limitations

To complement the quantitative evaluation, we further analyze conditions under which the proposed framework may experience performance degradation or operational limitations.

Although the proposed hybrid framework demonstrates strong adversarial robustness, several potential failure scenarios should be considered. First, the residual-based adversarial detector relies on reconstruction discrepancies produced by the denoising autoencoder. Extremely low-magnitude or highly optimized perturbations that preserve reconstruction consistency may bypass detection while still influencing classifier decisions. Such attacks can occur when adversarial perturbations lie close to the learned data manifold.

Second, performance may degrade under significant distribution shifts, such as network environments with traffic patterns that differ substantially from the training dataset. Since the model learns statistical characteristics of specific traffic distributions, unseen protocols or evolving attack behaviors may reduce detection reliability without periodic retraining.

Third, adversarial training improves robustness but introduces a trade-off between computational cost and scalability. Generating adversarial samples during training increases optimization time and resource consumption, which may limit deployment in real-time or resource-constrained environments.

Fourth, borderline traffic flows that share overlapping statistical properties between benign and malicious classes remain inherently difficult to classify. In such cases, even robust models may exhibit uncertainty, leading to occasional misclassification or rejection decisions.

Finally, adaptive attackers aware of the detection mechanism could attempt to jointly minimize classification loss and reconstruction residuals, potentially reducing detector effectiveness. Addressing such adaptive threat models represents an important direction for future research.

Building upon the previously discussed failure scenarios and limitations, we now discuss the broader practical implications and future research directions of the proposed framework.

### 5.4 Practical implications and future directions

The implications of these results extend to real-world deployment scenarios where deep learning models face adversarial threats in safety-critical domains such as healthcare, finance, and autonomous systems. The observed decline in calibration error across training epochs is particularly significant, as it indicates that the model not only becomes more resistant to adversarial manipulation but also more reliable in its probabilistic predictions. Improved calibration reduces the risk of overconfident errors, thereby increasing the trustworthiness of decision-making systems deployed in high-stakes environments.

From a computational perspective, the stabilization of runtime per epoch demonstrates that the proposed framework remains efficient despite the inclusion of adversarial training and residual-based detection. This suggests that enhanced robustness can be achieved without incurring prohibitive computational overhead, supporting the scalability of the approach to larger datasets and more complex architectures.

Despite these encouraging results, several limitations remain. The current evaluation is restricted to gradient-based white-box attacks, and robustness against adaptive black-box strategies, AutoAttack-style ensembles, or physical-world perturbations has not yet been explored. In addition, while the framework performs well on the evaluated benchmarks, its scalability to extremely large-scale datasets and high-dimensional domains such as medical imaging warrants further investigation. Finally, although the proposed approach narrows the trade-off between robustness and clean accuracy, it does not completely eliminate it, indicating room for further refinement. In parallel with these limitations, recent research has also explored defensive frameworks specifically designed to improve robustness of machine learning-based network intrusion detection systems under adversarial settings. For example, Tafreshian and Zhang [33] proposed a defensive framework that integrates adversarial resilience mechanisms directly into the IDS pipeline to mitigate evasion attacks. More recently, Barik and Misra [34] introduced a comprehensive defense approach that combines multiple robustness-enhancing strategies within deep learning-based NIDS to improve resistance against adversarial perturbations while maintaining detection capability. While such approaches demonstrate improved protection against crafted attacks, they primarily emphasize robustness enhancement and attack mitigation without explicitly addressing calibration behavior or generalization stability. In contrast, the proposed framework jointly considers robustness, calibration reliability, and generalization performance, providing a more unified perspective for trustworthy intrusion detection.

Looking ahead, future work may extend the framework to multi-attack training regimes, integrate it with emerging architectures such as vision transformers and graph neural networks, and explore complementary objectives including fairness, interpretability, and energy efficiency. Investigating real-world deployment case studies—such as adversarial robustness in healthcare diagnostics or financial fraud detection—will be essential to validate the practical impact of the proposed approach in complex operational settings.

In summary, this work advances the state of the art by demonstrating that adversarial robustness, generalization, and calibration can be jointly improved within a unified learning framework. While challenges remain, the proposed method provides a strong and extensible foundation for the development of resilient and trustworthy machine learning systems.

## 6 Conclusions

This study introduced a novel adversarially robust learning framework that integrates adaptive adversarial training with stability-oriented regularization to address the trade-off between robustness and generalization in deep learning models. The proposed method demonstrated the ability to withstand strong white-box attacks while preserving high clean-data accuracy and improving calibration, which collectively emphasize its practical applicability to safety-critical domains where reliability and trustworthiness are essential. Through the analysis of training dynamics, gradient norms, and calibration error trends, the work provided deeper insights into how robustness evolves over training and how the proposed strategy balances resilience with generalization. Beyond performance gains, the novelty of this framework lies in showing that robustness and calibration need not be competing objectives, thereby offering a new pathway toward trustworthy AI systems that are both reliable under attack and dependable in their probabilistic outputs. At the same time, the study acknowledges several limitations, including the scope of adversarial evaluations being restricted to gradient-based attacks, the absence of validations on very large-scale datasets, and the lack of exploration of other dimensions of trustworthiness such as fairness and interpretability. These limitations point to future directions where the framework can be extended to multi-attack and multi-modal settings, adapted to emerging architectures like transformers and graph neural networks, and optimized for deployment in resource-constrained environments. In addition, coupling adversarial robustness with complementary goals such as energy efficiency, fairness, and interpretability represents a promising area for further exploration. In conclusion, this work provides a unified approach to achieving robustness, generalization, and calibration in adversarial learning, laying the foundation for the development of machine learning systems that are not only secure and efficient but also trustworthy and scalable for real-world, high-stakes applications.
